# Coaches’ Mind Games: Harnessing Technical Fouls for Psychological Momentum in Basketball

**DOI:** 10.3390/bs13110904

**Published:** 2023-11-02

**Authors:** Gershon Tenenbaum, Ady Vigodsky, Assaf Lev

**Affiliations:** 1 B. Ivcher School of Psychology, Reichman University, Herzliya 4610101, Israel; gtenenbaum@admin.fsu.edu (G.T.); ady.vigodsky@post.runi.ac.il (A.V.); 2 Department of Sports Therapy, Ono Academic College, Kiryat Ono 55000, Israel

**Keywords:** basketball, technical fouls, psychological momentum, coaches, emotions, cognition, qualitative research

## Abstract

This study explored the emotional and cognitive dimensions associated with technical fouls (TFs) in basketball coaching. Using in-depth interviews with Israeli first-division basketball coaches, we aimed to uncover the emotional and cognitive intricacies involved in TFs. Through rigorous thematic content analysis, we delved into coaches’ ability to perceive and manipulate psychological momentum on the court. We revealed that coaches intentionally use TFs as strategic tools. TFs were employed to elicit specific emotional responses and cognitive shifts in players, affecting their emotional states, arousal levels, motivation, and overall team performance. However, coaches approached TFs with caution due to the associated emotional and cognitive risks. They carefully weighed potential benefits against unforeseen consequences in their decision-making. Furthermore, our research highlighted coaches’ belief in the immediate positive impact of TFs on referee decisions, underscoring the interplay between emotional influence and strategic advantage. Nevertheless, this advantage was perceived as short-lived, emphasizing that TFs are a two-edged sword with unpredictable outcomes.

## 1. Introduction

In the realm of competitive basketball, the dynamics on the court extend beyond the physical skills of players and the rules of the game. They encompass a complex interplay of social interactions, strategies, and symbolic gestures that come together in an emotionally charged performance. In this reality, basketball coaches are central actors, influencing the course of the game through their actions and interactions. The basketball court itself assumes the role of a grand stage, replete with an audience comprising players, referees, fervent fans, and fellow coaches, all of whom bring their own emotions and cognitive processes into the mix. In this “front region of behavior”, as Goffman [1] aptly described it, technical fouls (TFs) assume a pivotal role, emerging as symbolic acts laden with the potential to disrupt, reshape, or even heighten the performance of the actors. In basketball, a TF assigned to a coach is a penalty issued by the officials for unsportsmanlike conduct or any rule violation committed by the coach. This encompasses actions such as taunting, throwing objects, or inciting the crowd. If a coach accumulates two TFs in a single game, he/she will be ejected from the game. It should be noted that technical fouls can carry repercussions for the team, including awarding the opposing team free throws and potentially resulting in the coach’s removal from the game.

A common categorization within the concept of momentum distinguishes between personal momentum and team momentum. Personal momentum is tied to the “hot hand” effect, a belief that when a specific player consistently makes consecutive shots, he/she is likely to maintain their streak due to being “hot”. This concept is predominantly associated with basketball, and it is evident that many coaches and players adhere to it, often consciously choosing to pass the ball to the “hot” player [2,3]. The focus of this study is on team momentum. Team momentum refers to an enhancement in the performance of an entire team during a single game or over a series of games, irrespective of whether the team is on a winning or a losing streak [4,5]. Accordingly, this study embarks on an exploration of how basketball coaches harness technical fouls as a strategic tool for altering the psychological momentum within a game, tapping into the deep well of emotions that permeate the court. Despite the analytical challenges in pinpointing momentum, coaches and fans commonly work under the shared belief that momentum not only exists but can also be interrupted [4]. In this reality, the study aims to dissect how coaches strategically deploy technical fouls to influence the game’s momentum, taking into account not only their own emotional states, but also their understanding of their players’ and opponents’ cognitive processes. More specifically, the study sheds light on the strategies that may precede these performances, uncovering the practices and preparations that may occur before a coach commits a TF.

In delving into the emotional and cognitive aspects of this phenomenon, we seek to reveal the psychological chess match that unfolds alongside the physical contest. It becomes apparent that coaches must navigate a complex landscape of emotions, from frustration and anger to strategic calculation, all while considering how these emotions might affect the cognitive processes of their team and their opponents. Thus, momentum appears to evoke substantial emotional, behavioral, and cognitive responses, giving rise to discernible patterns of reactions to competitive events. These responses, displaying distinct variations among players and coaches, unfold as performance assessments and are likely to have significant implications for the individuals undergoing them [6]. All in all, the present study ventures beyond the conventional analysis of rule violations, delving into the role of technical fouls as symbolic acts that reverberate through the social fabric of basketball games. In doing so, we aim to shed light on the hidden dimensions of coaching in basketball, offering a fresh perspective on the intricate dance of sportsmanship, strategy, and social performance in reference to TFs. This exploration promises to unveil the deeply intertwined emotional and cognitive facets of this captivating aspect of the game.

### 1.1. Psychological Momentum in Sports

Studies on psychological momentum in sports often fail to provide an unequivocal answer regarding its significance. Many struggle to find evidence of psychological momentum in sports, and when a clear momentum impact is discernable, it is often of low intensity. More specifically, psychological momentum in basketball is an elusive, fleeting phenomenon, and one that is difficult to define [7]. However, although momentum is challenging to generate, it holds a significant value for both athletes and coaches [6]. On the whole, the concept of momentum influences coaches to strategically implement actions when the team is experiencing the momentum (e.g., on a scoring streak) of consecutively scoring several baskets, while the opposing team, on the other hand, scores few to none [8,9].

Adler and Adler [10] were the first to define psychological momentum as a particular behavior that leads to the same behavior or to success, gives rise to additional success, or increases the chance of a positive result. Creating psychological momentum in sports is manifested in achieving a psychological state that affords the athlete a sense of superiority over his/her opponent [11]. This elevates a sense of self-efficacy [12], which ultimately affects performance. Vallerand et al. [13] defined psychological momentum in sports as a positive or negative change in one’s physiological and emotional state, caused by a preceding event or a series of events, and which leads to a change in the performance outcome. His model postulates that psychological momentum consequences are moderated by the context of the specific situation and the players’ characteristics and abilities.

Psychological momentum is more pronounced immediately following the preceding event (e.g., a technical foul in basketball) and was not found to be associated with final performance outcomes [14]. Taylor and Demick [15] recognized that psychological momentum does not necessarily manifest itself in long-term results but rather has immediate effects. They introduced the notion of a “momentum chain”, in which game momentum is precipitated by an event or events manifested by changes in the players’ emotional and behavioral state and performance ability, in their opponents’ psychological states, and in outcome indicators. Crust and Nesti [12] argued that a precipitating event and the immediate outcome modeled by Taylor and Demick [15] do not accurately reflect the entire scope of psychological momentum in sports, and that further studies on the perception of momentum are required.

Using in-depth interviews with professional soccer players about their perceptions of psychological momentum in sports, Jones and Harwood [16] identified four categories associated with the phenomenon: experiences of positive momentum, experiences of negative momentum, strategies for developing/maintaining positive momentum, and strategies for overcoming negative momentum. Within the positive and negative momentum experiences, these researchers distinguished between triggers and outcomes. For example, an error conducted by a team may trigger a positive momentum for the other team. Such shifts in psychological momentum are manifested in changes in individual and team psychological processes such as self- and team confidence, mutual encouragement, communications, and shared mental models.

### 1.2. Psychological Momentum in Basketball

In the research literature, very little has been devoted to the context of TFs in basketball. In one study, Gomez et al. [17] examined the immediate impact of TFs on the performance of the team committing the TF and the performance of the opposing team. The authors studied the impacts of TFs called on bench personnel and coaches in Olympic games, European championships, and world championships. The results showed that when TFs were assessed against coaches/bench personnel, the negative momentum of the losing team during the game halted. This research finding suggests that coaches are aware of when their teams are in a decline, and intentionally commit TFs to influence the referees’ perception and change the game’s momentum. In another study, Zitek and Jordan [18] found that instrumental aggressive behavior in the NBA, as coded by the number of TFs awarded, was generally associated with higher success rates in games. For example, NBA players who received more TFs scored more points and accumulated a higher number of rebounds and blocks. Furthermore, in a recent study by Weimer et al. [4], new evidence has emerged indicating the presence of team-level momentum in the NBA. The researchers conducted an analysis of momentum dynamics at the team level, with a particular emphasis on the influence of TV timeouts on momentum. They employed TV timeouts as a natural experimental setting to gauge the consequences of game interruptions on momentum. The study revealed that TV timeouts lead to an 11.2% decrease in the points scored by the team holding the momentum. Importantly, this effect remained consistent and was not influenced by factors such as the length of a scoring streak, player’s substitutions, or the overall game context.

Moreover, considering the fact that a portion of the effort to make an action that affects the game’s momentum, which involves performance improvement and change, the critical standpoint of Goffman [1] is regarded as a critical cultural analysis in sports [19]. It provides a solid conceptual framework for the study of the outcomes of technical fouls by coaches. According to Goffman, social interaction, such as the interaction between referees and coaches, is a stage where the coach aims to alter his/her perceptual management over his/her players, and therefore change the momentum of the game. However, it must be noted that coaches encounter numerous challenges when attempting to foster momentum. In this context, Schoen [6] argued that cultivating a team-oriented approach becomes increasingly arduous in a sports culture that emphasizes individual excellence. Coaches frequently express their frustration with the contemporary difficulty of nurturing trust among players and helping them overcome egocentric comparisons with their peers. Furthermore, Schoen highlighted the apparent diminishing capacity of younger players to attentively listen to and comprehend coaching instructions, particularly during intense moments in a game when emotions intensify. Therefore, one of the crucial prerequisites for momentum to manifest is the presence of emotionally intelligent and mindful floor leaders.

The present study seeks to capture the perceptions of psychological momentum when a TF is committed either spontaneously or intentionally by a coach during an episode of negative momentum. Questions of relevance in the current study relate to (a) *pre-event—*the preliminary phase in which the negative momentum emerges, leading the coach to engage in unsportsmanlike behavior; (b) *the event—*the phase immediately after a TF/coach ejection occurs, when the game stops for several minutes, and the elevated emotional arousal of the players, the audience, and the referees triggers an emotional contagion among the players on the court, which leads to a change in the players’ motivation and perception of their team’s competence; and (*c) post-event—*players return to the game with elevated emotional arousal and perception of team competence.

As far as we know to date, there is a lack of evidence concerning the effects of coaches’ TF on psychological momentum in sports, and its associated players’ emotional contagion, motivation, and perception of team competence. We addressed this void by conducting a qualitative study in which elite coaches were interviewed and their reflections were analyzed. Specifically, we examined whether professional basketball coaches perceive TFs as an effective method for reversing negative psychological momentum, and whether they intentionally use TFs for this purpose. The following general research questions are explored: (a) Do professional basketball coaches intentionally use TFs to stop negative momentum? (b) Do coaches perceive TFs as an effective method for changing their players’ psychological state? (c) Do TFs affect a referee’s decision-making (DM)?

## 2. Method

A qualitative study was undertaken using the grounded theory approach, as outlined by Glaser and Strauss [20], to explore the motivations of basketball coaches for committing technical fouls (TFs). This approach seeks to elucidate behavioral phenomena by thoroughly analyzing data acquired from field observations and interviews that encapsulate the phenomenon under investigation.

### 2.1. Participants

Participants were seven Israeli male basketball coaches with at least five years of coaching experience in one of the two top Israeli teams (e.g., the Premier League and the National League) or a national team. The coaches were selected in a convenience sampling method. The coaches’ ages ranged from 40 to 66. Six currently live in Israel and one lives in Europe. Three of the interviewees coached a Middle Eastern national team over a period of years: One was head coach of an Israeli male national team for four years, and two were head coaches of the Israel’s female national team. Additionally, two interviewees coached in the Euroleague as part of their professional careers, and one won the Israel’s first division championship and three championships in a European league. Three were coaches of various European clubs and two coached European national teams. Six of the seven interviewed coached in Israel’s Premier League. The coaches in the study were assigned fictitious names for reasons of confidentiality.

### 2.2. Procedure

The research proposal was submitted to the institutional review board. Following the committee’s approval, the coaches were contacted by phone and enrolled in the study. Before conducting the interviews, the coaches’ career history was reviewed (e.g., the teams they coached, significant career achievements, coaches who influenced them). Individual interviews were conducted in person, and lasted about 90 min. The interviews were recorded and verbally transcribed. The transcribed interviews underwent a content analysis to identify central themes related to the research topic.

Semi-structured interviews (SSI) were conducted to extract novel insights that emerged from the interviewees’ narratives, and the meanings they attributed to their first-hand experiences on the court. SSIs encourage participants to bring up anecdotes that reveal the importance and context of events, as well as participants’ feelings and behaviors about them [21]. Prior to the beginning of each interview, the coaches were briefed on the study’s aims and method and signed a consent form expressing their agreement to participate in the study. The form, which included a brief explanation of the study, stated that the study was approved by the University’s ethics committee, ensured the participants’ anonymity, and elaborated on their rights*—*including their freedom to choose not to participate in the study or to leave it at any time without any consequences.

Interviews touched on the following issues: (a) Pre-event*—*the preliminary phase in which the negative momentum is created, leading the coach to conduct unsportsmanlike behavior (e.g., how did you identify the evolution of the momentum, what was the moment that you felt that there was psychological momentum?); (b) the event (the TF; e.g., “What was your reason for committing a TF?”); (c) post-event*—*a return to the game with a marked change in emotional arousal and perceived team competence (e.g., “Did you sense a change on the court following the TF?”); (d) emotional contagion, motivation, and perceived team competence (e.g., “Please describe the TF’s immediate impact on the players of your team.”).

### 2.3. Content Analysis

The content analysis identified the primary, secondary, and tertiary themes concerning professional and experienced basketball coaches’ use of TFs and their perception of the TFs’ impact on a game’s psychological momentum. The analysis followed Malterud’s [22] Systematic Text Condensation (STC) qualitative content analysis approach. Its starting point was Giorgi’s [23] method of analysis. Malterud [22] found STC to be an effective analysis method in qualitative studies based on interviews, observational studies, and written text analysis. In line with this method, interviews were analyzed by two experienced content researchers according to the following steps: After the transcripts were separately submitted to each of the researchers for analysis, each researcher read the transcripts in-depth, marking central repeating themes. The researchers then defined central themes by identifying a common denominator and a conceptualization uniting several content items dealing with the same topic. The researchers compared the central themes to find common themes. The interview transcripts were reanalyzed to confirm the central themes identified by the researchers. Finally, central themes were examined in light of the assumption that professional basketball coaches perceive TFs as an effective tool for reversing negative momentum and creating positive momentum. It should be noted that this procedure was iterated until a final list of themes representing the data was determined by consensus.

### 2.4. Quality Assurance

To ensure quality and trustworthiness, the study design implemented Tracy’s [24] eight quality criteria for qualitative methods across universal models. Applicable theoretical foundations were utilized to meet the study’s objectives, methodology, and findings. Distinct inclusion guidelines were followed to ensure that the selected sample pertained to the study’s objectives. To establish the credibility of the study, a process of triangulation [25] was performed. Here, the principal and additional investigator carefully read the transcripts, after which a consensual approach was taken to study the findings and resolve differences among the researchers in order to attain a final coherent version of the general themes and the associations among them. The current study offers a significant theoretical contribution to the existing literature by probing an under-studied issue regarding the effect of TFs committed by basketball coaches on the game’s psychological momentum, and how experienced basketball coaches perceive their own use of TFs.

## 3. Results

**Figure 1:**
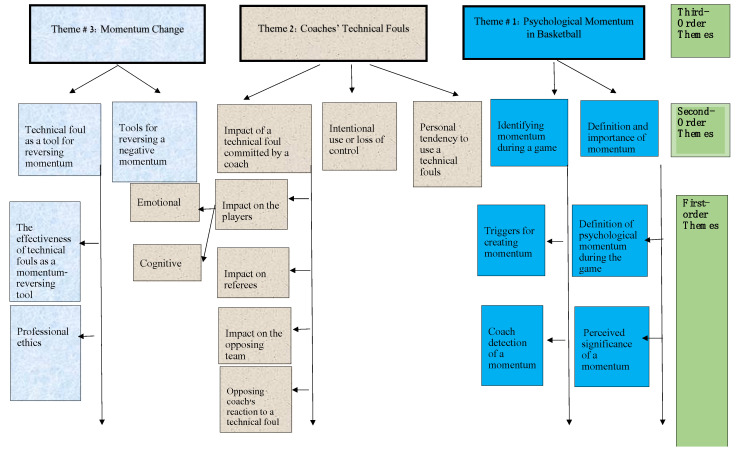
Higher and lower-order themes generated from Interviewing the basketball coaches.

The data revealed three high-order themes containing sub-themes (see Figure 1). The first high-order theme refers to coaches’ perceptions of psychological momentum and its significance. The second high-order theme pertains to coaches’ use of technical fouls (TFs) and the rationale behind their behavior (offered by the interviewees). The third high-order theme refers to coaches’ perceived ability to change psychological momentum, and specifically the tools available to enable the coaches to do so.

### 3.1. Theme #1. Psychological Momentum in Basketball

#### 3.1.1. Definition and Importance of Momentum

*(1)*  *Definition of Psychological Momentum during the Game*

All seven coaches defined psychological momentum in basketball in a similar manner: the ability of a positive action or a series of actions on the court to create a positive change in the players’ *emotional state* (e.g., enthusiasm, confidence, feeling of competence), leading to sustained positive performance. For example, one of the coaches, Adam, defined momentum as:

*“A good action or a collection of good actions by an individual or the team, bringing about a peak of enthusiasm and confidence.”*

Another coach, Sam, agreed, adding:

*“You feel a sudden increase in confidence, adrenaline, enthusiasm, and usually, an enhanced level of performance.”*

Expanding briefly on the coach’s role in his definition of momentum, Tony stated:

*“In a series of positive activities, in defense or offense, the players believe in the coach and his/her work with them, creating a process of momentum in the game.”*

A fourth coach, John, elaborated on a definition which underscored the positive effects of momentum:

*“It’s a sequence of decisions and actions that are not always recorded by statistics. I feel it’s one of the most meaningful events in the game and a motivating factor for the team, which builds some momentum. Another element is team coordination; you see a team that is psyched in a good, defensive way—a kind of ecstasy, reaching a phenomenal level of enthusiasm.”*

*(2)*  *Perceived Significance of a Momentum*

The coaches interviewed in the current study perceived the psychological momentum during the game as one of its most critical factors. They viewed basketball as a game in which the momentum moves from one side to the other, and where momentum is considered to be an event that produces a significant advantage. For example, John stated:

*“How vital is psychological momentum during a game? It’s like asking how important it is to breathe (laughing).”*

Sam described the perceived significance of momentum:

*“The most dominant characteristic of the game is changing momentum. It’s most likely that the team that succeeds in generating momentum and maintaining it over time, and is the fastest in stopping negative momentum, is the better team.”*

#### 3.1.2. Identifying Momentum during a Game

*(1)*  *Triggers for Creating Momentum*

According to the coaches interviewed in this study, the trigger is an event or sequence of events that starts the momentum chain. The coaches described different events as possible triggers of momentum. For example, the following actions on the court were singled out by Brent: “*6–0 run, two, three turnovers, two 3-point shots in a row*”. More broadly, Sam explained:

*“Triggers are turning one mistake into two mistakes... meaning that we missed a layup and, out of frustration, committed an unsportsmanlike foul on the way to defense; usually, the second and third actions produce the negative momentum”.*

When further elaborating on momentum triggers, Adam argued that even a single extraordinary action can generate momentum. He stated:

*“It could be a stop in the lane, a charge, a big rebound, a big steal. In offensive situations, we talk about a big basket, a big pass, a changed outcome, a change in the situation where you were chasing a team, and suddenly you shoot a basket, and finally you lead”.*

*(2)*  *Coach Detection of a Momentum*

The importance coaches attribute to momentum underscores the need to identify momentum correctly in order to respond effectively. For example, John suggested:

*“Recognizing momentum comes first, and then you can make some tactical moves, and like everything else, sometimes it goes great, and sometimes you see it’s not helpful”.*

In underlining the significance of a coach quickly identifying situations during the game that could rapidly shift the momentum, George stated:

*“I feel it in the players’ vulnerable moments—in small crises, when someone fails to return to defense or fails to close the base-line. Simple elements that you can examine and observe. If you observe these, the crisis [will be] small”.*

Expanding on George’s point, Tony explained that the coaches mainly look at the players’ various behaviors to identify momentum:

*“Body language and speech. The players’ behavior toward each other, toward you. Many more positive things. When there is no momentum and you replace a player, the one who is replaced is always dissatisfied, which makes sense, but when momentum is positive the players understand that that’s how the system works, so it’s much easier for them to accept things”.*

Another coach, Brent, reiterated Tony’s point:

*“Body language gives us a lot. Counting the ‘high-fives’ exchanged by the players is a common sign”.*

### 3.2. Theme #2. Coaches’ TFs

#### 3.2.1. Personal Tendency to Use a TF

Most coaches claimed that they are not inclined to use this maneuver frequently. In their opinion, TFs may have advantages, but they also carry risks, which might be reflected in loss of control over the game, negative effects on a specific player or the club spirit, or destructive internal and external communications (with the referees and the other team). The coaches clearly stated their preference for more ethical tools, as Sam said:

*“I’m not a big fan of TFs. I’m always more concerned about their consequences because while I may have gained a momentum change, how my players perceive me is also important; legitimacy is essential. I realize TFs can help change momentum, but they may give my players permission to lose their tempers or talk inappropriately to the referees”.*

Adam also explained:

*“I’m not the type of person who tries to get a TF to change the momentum in a game; I don’t believe in that. And I would certainly not do that at the high levels”.*

In a different vein, preferring to lean to the positive side of the game, Brent stated:

*“We have a positive attitude (toward the game), we have passion and fun. That’s what I believe in, and that’s the spirit we discussed—the positive spirit and the positive psychological mindset we need to create. I don’t get many TFs because I’m also on good terms with the referees”.*

#### 3.2.2. Intentional Use or Loss of Control

The coaches were almost unanimous in their opinion that most TFs are committed by coaches deliberately in order to influence the course of the game. The following four coaches all provided further clarity in this regard. For instance, Sam said:

*“20% of coaches’ TFs are outbursts, while 80% are under control. Some coaches have an inherent agenda to badger the referees”.*

Similarly, Tom stated:

*“The coach knows he’s getting a TF, he knows what he’ll gain from it. The question is, when you get a TF—is it your ball or the opposing team’s? It’s an art”.*

Brent concurred, adding:

“The majority [of TFs] are intentional, a lot of it is show, a lot is acting”.

Adam offered an example of what he often does prior to receiving a TF:

*“I can say that I lost control very few times. I often turn to my assistant and say ‘Everything is under control, don’t worry’. I received TFs, but none occurred because of a lack of control”.*

#### 3.2.3. Impact of a TF Committed by a Coach

A TF committed by a coach is an unusual event, which impacts on a wide range of variables. Here are the coaches’ reflections on the impact of TFs.

*(1)*  *Impact on the Players*

When a coach tries to stop the game and change the players’ emotional state by committing a TF (emotional impact on the players), it may be viewed by the players as a loss of control by the coach or a breach of professional boundaries (cognitive impact on the players), which affects them negatively. Most coaches pointed out that the effect of a TF on players depends on the player and the situation. Thus, it is crucial to commit a TF in the proper situation, with a composition of players who will respond positively. John illustrates the complex nature of a TF’s effects:

*“What works for player A will not work for player B, and what works for player A on a certain day will not always work for him on another day. Thus, TFs depend greatly on the situation, and I can’t define them”.*

- *Emotional*

The coaches believed that a TF made by a coach may result in elevated alertness levels for some players and could trigger emotional contagion that affects the entire team. Coaches perceive a rise in alertness as a necessary step in their efforts to change a game’s momentum. George explained this shift in alertness as follows:

*“It produces a change in the energy of the team. Say we count on a scale of 1 to 10, and the energy was a 5—it will move to either an 8 or a 2, depending on the players. Some may break and feel shaken up if it’s something they’re used to having relatively in control”.*

John adopted a wider perspective, stating:

*“You evaluate the situation and see that some players are somewhat apathetic and lethargic. First you try to go about it directly and shake them up, and when you see it’s not working, then maybe the shock caused by a TF can succeed in stirring them up”.*

- *Cognitive*

In contrast to the temporary emotional effect of TFs, coaches believed that a TF has a negative cognitive impact on players that may extend over a long interval, possibly over the remaining duration of the game. The coaches were concerned by being viewed by players as having “lost control” or permitting unsportsmanlike behavior. Sam shared his concerns:

*“I’m always more concerned by the broad consequences, because although I may have gained a change in momentum, there is also significance in how I am perceived by my players. Acting legitimately is no less important. I know a TF may help change the momentum, but it may also allow my players to lose control or to talk unacceptably to the referees”.*

Referring to players’ personalities, Tom stated:

*“I find this question difficult to answer because it impacts each player differently. One might wonder, ‘what is this idiot doing’? We’ll lose momentum because of him. It depends on how well you know your team”.*

Adam similarly added:

*“It affects each player differently, some don’t like it. They look at you and ask, ‘what the hell are you doing”.*

*(2)*  *Impact on Referees*

Most of the coaches believed that a TF may make referees feel obliged to balance the whistles for the TF with whistles against the opposing team, although the coaches agreed that this effect, if it occurs, is brief and depends on the referee’s skill and experience. Regarding the potential psychological impact on a referee, Brent said:

*“Psychologically, it may (affect) a referee, who will then whistle a little in my favor; it can’t hurt”.*

Adam explained that the referees’ experience may affect their subconscious handling of a TF:

*“[It] depends on the skill level of the referee. If the referee’s level is high, there is not much significance. At the lower skill levels, it may have some”.*

John elaborated on the risk element:

*“The journalists will be excited, ‘That TF was genius,’ but you don’t know where it’s going. You make a risk assessment, and sometimes you have nothing to lose”.*

Finally, George stated:

*“It works on the referees too. Some will say they ignore it, but I say there’ll be whistles in your favor, two-and-a-half, three minutes after the event”.*

*(3)*  *Impact on the Opposing Team*

The coaches also addressed the potential consequences of a TF on the opposing team, although the coaches were divided on the nature of this effect. Brent explained:

*“First, the other team mellows; they say, ‘wait, let’s stop’. It affects them as well. The other coach suddenly becomes cautious because he says, ‘just a minute, he received a TF, they will want to get back at us’. There’s a psychological effect where he says, ‘there’s going to be payback’. So he suddenly takes a step back”.*

In contrast, Sam stated

*“In general, I’d say it also gives the opponent energy”.*

*(4)*  *Opposing Coach’s Reaction to a TF*

The responses of the opposing coach are directed, first and foremost, toward the referee and the players of the TF offending team, typically to reduce their level of excitement and the effect that the TF was designed to create. TFs are typically not ignored. All the coaches stated that they would respond to a TF committed by the opposing coach, which indicates the significance they assign to a TF. For example, Adam said:

*“My first reaction? Watch out; the referee will have his eyes on us. That’s it”.*

Brent agreed and expanded on Adam’s statement:

*“I respond. I can’t ignore it. First, I say to the referees, ‘Hey, listen, he committed a TF,’ and I’ll say it out loud so he can hear it too, and everyone can hear it, my players and the crowd. It’s psychology, so I do it vocally and theatrically”.*

Tony further explained how a coach may influence a referee and guide his players:

*“Extinguish the situation. If the referee comes, I say, ‘What is he thinking, tell me, now you’ll start whistling against me?’ Then I say to my players, ‘Notice how the next two whistles go to the other team. Don’t get over-excited; I’m talking from experience’”.*

Sam stated similarly, *“I tell my guys, ‘okay, he’s trying to do something here, let’s be even stronger together’”.*

### 3.3. Theme #3. Momentum Change

#### 3.3.1. Tools for Reversing a Negative Momentum

The coaches perceived psychological momentum as one of the most influential factors to a game’s outcome. Almost all of them mentioned the need to stop the game in the event of negative momentum and change their players’ emotional and motivational state. The coaches feel they have a wide range of tools to change the momentum during a game, and this toolbox includes TFs, which both interrupt a game and change players’ psychological states. In this context, Brent stated:

*“It could be a substitution of two players that suddenly stirs the team. It could be a timeout that stops the negative psychological momentum. It could be a deliberate TF, but then the team takes it hard, saying, ‘wait, something happened here’, so we sometimes even pour water on the court to stop the game for a moment. We even tell a player, ‘you’re injured, lie down’, to pause the game. It’s a bit extreme and theatrical, but you want to stop this thing for a moment”.*

Similarly, Tony offered examples of a coach’s tools to stop a game:

*“Everything, from changing the defensive and offensive tactics, substituting a player, taking a timeout. A coach has ways to stop the game”.*

Ron further replied:

*“I’ll do anything to stop the game when needed, even extreme action. For example, for me, extreme action is to attack the referees, or, and I’ve done this more than once in my life, to grab my player and shake him until blood comes out of his ear. It’s taking my number one player and pressuring him. I mean, [doing] something unpredictable”.*

Another coach, George stated:

*“If I see a snowball effect of negative momentum, I immediately take a timeout. If I think a substitution can stop it, or a TF can stop it, if I think that a word with the referee, or something that disrupts the pace of the game will stop it, or if I can tell a player to untie his shoelaces, we do that too”.*

#### 3.3.2. TF as a Tool for Reversing Momentum

*(1)*  *The Effectiveness of TFs as a Momentum-Reversing Tool*

Although the coaches agree that TFs are committed by coaches deliberately and are usually not the result of an emotional outburst, their opinions vary regarding TFs’ effectiveness to change the momentum during a game. Four of the seven coaches believe that TFs are effective, while the remaining three believe that a TF has no significant effect. John explained:

*“It does make a difference. Players who care, feel connected to the team, and aren’t among the few indifferent and apathetic individuals, are affected. It does make a difference”.*

George similarly argued:

*“Why would you commit a TF? To change the situation, to change the momentum. You don’t do it just to gain two or three whistles in your favor. First, you stop the momentum. You want the momentum change to impact the referee, change the game, change the pace, so you do it, you initiate it. From 1 to 5? I would give it a 3.5–4 that it works”.*

In contrast to John and George, Sam, one of the coaches who believes that TFs have little if any impact, stated:

*“Overall, I think it’s somewhere between a neutral and a small positive effect. There may only be some small effects. I’ll be surprised if you bring about a meaningful change”.*

Agreeing with Sam, Ron said: “*The ultimate effect of a TF on the game is not good*”.

*(2)*  *Professional Ethics*

Collectively, veteran coaches do not advise young coaches to use TFs. Even the coaches who asserted that TFs are effective in reversing a game and/or changing momentum do not recommend using it at the beginning of one’s professional career, mainly because its effectiveness depends on many factors, requiring extensive experience to be used effectively. For example, Adam said:

*“As a young coach I wouldn’t use anything related to TFs as a method. I don’t think that it should be used systematically. Let the players understand that something unusual happened when you got a technical foul. But you must make them understand that you haven’t lost control”.*

Tom concurred and was even more decisive:

*“No [as a recommendation to a young coach]. Start coaching, start working, and don’t start managing the game. Be strong, promote the kids, and work hard. Don’t get involved in all this nonsense; it’s not suitable for youngsters”.*

John’s recommendation for a young coach was:

*“I would tell him to try to focus on the essence. Try not to use a TF, only when you feel you have no choice”.*

## 4. Discussion

The present study examined how basketball coaches perceive technical fouls (TFs) as an effective method for reversing the psychological momentum and unfolding events of a game. The content analysis of interviews with seven expert basketball coaches indicates that basketball coaches define psychological momentum as a positive action, or a sequence of actions, on the court that creates an immediate change in the emotional and cognitive state of the players on the court (e.g., heightened enthusiasm, confidence, and sense of competence), which leads to positive, mainly short-term, performance outcomes. At the same time, the coaches reported that the opposing team players experience a decrease in all these indicators. Accordingly, the coaches in our study believe that a TF committed by a coach leads to an immediate change in a game’s psychological and technical mechanisms but does not necessarily manifest in long-term effects. This finding supports Vallerand et al.’s [13] and Taylor and Demick’s [15] conceptualization. Furthermore, the findings align with Gomez et al.’s [17] observation that the most substantial impact of technical fouls on outcomes occurs in the short term, particularly when the technical foul is committed by the coach instead of a player. A consensus among the basketball coaches who participated in this study is that psychological momentum is one of the most critical strategies used to influence the game’s sequence, although the coaches do not unanimously agree about implementing it during a game.

According to the primary psychological momentum model [13] and the momentum chain model that refers to the immediate impact of momentum [15], psychological momentum is triggered by an event, or a sequence of events, and is regarded as one which may positively change the sequence of unfolding events. Findings of the current study support the significance of psychological momentum in a game and its identification. The coaches in our study agreed on the importance of identifying and distinguishing events that generate momentum from those which fail to do so. The capacity to properly identify the momentum-generating event requires experience and skill and is essential for the coach to manage the game. Early identification allows the coach to react faster and reduce the damage to his team. All in all, these results echo the conclusion of Schoen [6], maintaining that coaches who can cultivate emotional awareness and effectively regulate their emotions may have a higher chance of achieving success in creating momentum by conducting an act such as a TF.

The content analysis shows that the coaches identify the players’ psychological state through subtle changes in the way their team members play: their players’ behavior, body language, and communications among themselves and with their coach. As one of the coaches described, *“Body language gives us a lot. It’s enough to count the number of high-fives players exchange”.*

Negative momentum is experienced earlier than positive momentum, and responses to negative momentum are more consequential [26]. When negative momentum is noticed, coaches recognize a decline in their players’ arousal and self-confidence accompanied by a decrease in their self-competence. One coach described the change as follows: “*I feel it in the players’ breaking points. In small crises, [like] when someone failed to return to defense, or failed to close the base-line. Simple elements that you can notice. Also, it doesn’t happen to one person alone; it’s contagious*. ”By identifying the changes in the players’ behaviors, the coaches identify the players’ emotional change. Therefore, an experienced coach must be perceptive and observant, e.g., “*feeling the players is perhaps the art of coaching. It is perhaps what differentiates a mediocre coach from a good one, a very good one, and an excellent one.*”

The findings also illustrate that the TFs coaches commit to stopping the game and changing the players’ level of cognitive and emotional states. The coaches’ reflections indicate that they intend using the TF to increase the players’ arousal level and motivations levels and, at the same time, to enhance their belief in their ability to change the momentum (e.g., “*We’re not as precise as we can be, we are capable of more*”). As an intentional act, a TF aims at influencing the course of the game through impression management [27]. The resulting change occurs through emotional contagion from the coach to some players, and from them to the entire team. This attempt aims to change the team’s dynamics [28]. As the findings show, the coach’s TF arouses the players’ emotions. Shared positive emotions increase team cohesion and identity and help in the efforts to achieve common goals [29]. However, coaches are concerned that some players might perceive the coach’s TF as illegitimate behavior, which may damage the coach’s status or give players the legitimacy to commit TFs themselves, which would be regarded as negative emotional contagion. In other words, along with the desire to generate a positive change, a TF committed by the coach carries a significant risk, which is the reason that coaches do not use this tactic indiscriminately. In this regard, the emotional and cognitive challenges involved in maintaining an effective impression management strategy demonstrate a form of “dramaturgical discipline”. As Goffman [1] (p. 17) aptly noted, the control of one’s facial expressions and voice serves as “the crucial test of one’s ability as a performer.” The coaches’ “face-work” is employed to ensure that their performance in front of both their players and the referees is respected and valued.

Furthermore, the study also examined the coaches’ perception of TFs’ impact on referees. The coaches believe that a TF produces a positive short-term effect, causing referees to become slightly biased in favor of their team in the immediate term. They believe that this bias stems from the referees’ tendency to “balance whistles” between the competing teams. These findings echo Anderson and Pierce’s 2009 study [30], which supports the referees’ tendency to balance the number of fouls for each team. Their study demonstrated that basketball referees whistle more fouls against the team with the lower number of fouls, and that the greater the difference in foul counts between the teams, the more likely the referees were to whistle against the team with the lower number of fouls.

Coaches feel that they have multiple means for reversing the momentum during a game, including player substitutions, timeouts, changing defensive and offensive tactics, proactively stopping the game, and committing a TF by the coach. The coaches view TFs as an effective means of stopping the game, changing the players’ emotional state, and even creating an immediate positive impact on the referee. Nonetheless, the findings indicate that the coaches did not fully agree on the effectiveness of a TF in changing the game’s momentum. Four of the seven coaches believed it had a certain positive effect, while the other three thought it has no significant effect. Some coaches perceived the TF as a less effective method because of the risk that the coach’s behavior might be perceived by the players as a loss of control or illegitimate conduct, which would give players a license to lose control. Basketball coaches are aware of the unique risks of using a TF, including the potential negative effects on the players in the immediate and long-term, and therefore prefer to use other methods to change momentum when possible.

## 5. Limitations and Future Research

The primary limitation of this study relates to the small sample size of experienced Israeli basketball coaches, which limits the generalizability of its findings. However, our conclusions consist of themes that were identified from a broad consensus among the interviewees. Quantitative research to explore how the themes identified in this study manifest in the field is warranted.

The themes identified in this study may be uniquely connected to aspects of Israel’s culture or specific characteristics of the Israeli basketball culture; other themes may be identified in other countries and cultures. A TF committed by a coach results from a deviation from his/her normal conduct during a game and requires a certain degree of aggressiveness, boundary-crossing, and audacity. These characteristics are culturally dependent and may vary across countries and cultures. More research in additional countries is required to explore the coaches’ opinions about the legitimacy and effectiveness of TFs committed by coaches in basketball.

Finally, psychological momentum exists in all sports. Therefore, all sports require effective methods to reverse and influence psychological momentum. This study centered on emerging themes raised by basketball coaches in connection with their use of TFs to reverse a game’s momentum. The study must be expanded to different sports and/or different basketball leagues in order to examine whether similar or new themes can be identified.

## 6. Conclusions

The current assessment of comprehending the dynamics of technical fouls (TFs) and their influence on athletic performance and psychological momentum in sports holds important significance. Its relevance is rooted in the captivating intrigue surrounding social factors that precipitate alterations in human and group behaviors. Our qualitative investigation, centered on TFs as a mechanism for reshaping the course of events, relied upon insights from highly experienced basketball coaches. They articulated their perspectives on this phenomenon and its perceived likelihood of success. We have discerned both the potential advantages and the pitfalls associated with employing TFs as catalysts for modifying emotional, motivational, cognitive, and team performance dynamics. However, we must investigate whether similar transformations can manifest in alternative physical and social contexts, and under what specific conditions.

## Data Availability

The data presented in this study are available in the presented paper.
